# Analgesic effect of the ultrasound-guided thoracolumbar paravertebral block in patients undergoing robot-assisted laparoscopic nephrectomy: a randomized controlled trial

**DOI:** 10.1186/s12871-024-02460-6

**Published:** 2024-02-22

**Authors:** Guojiang Yin, Yue Li, Pengxiao Wei, Xuyuan Ma, Bixi Li, Guosheng Gan, Xiaoyang Song

**Affiliations:** 1 Department of Anesthesiology, General Hospital of Central Theater Command of People’s Liberation Army, Wuhan, 430070 China; 2 The First School of Clinical Medicine, Southern Medical University, Guangzhou, 510515 China; 3 General Hospital Base of Central Theater Command of People’s Liberation Army, Hubei University of Medicine, Wuhan, 430070 China

**Keywords:** Ultrasound-guided, Thoracolumbar paravertebral block, Robot-assisted, Nephrectomy, Analgesic effect

## Abstract

**Background:**

Paravertebral block has similar effect as epidural anesthesia, and has good somatic and visceral analgesic effect. Paravertebral block is widely used in thoracic surgery, but rarely used in abdominal surgery.

**Aims:**

This study aimed to evaluate the analgesic effect of thoracolumbar paravertebral block in patients undergoing robot-assisted laparoscopic nephrectomy.

**Methods:**

One hundred patients undergoing elective robot-assisted laparoscopic nephrectomy were included in this study. Based on whether the thoracolumbar paravertebral block was performed, the patients were randomly divided into the thoracolumbar paravertebral block combined with general anesthesia group (TL-PVB group) and simple general anesthesia group (NO-PVB group). Oxycodone was administered for patient-controlled intravenous analgesia (PCIA). The primary outcomes included the amount of remifentanil used during surgery, the amount of oxycodone used in 24 and 48 h after surgery. Secondary outcomes included the changes of heart rate (HR) and mean arterial pressure (MAP), time for the first analgesia administration, visual analog score (VAS) of pain during rest and movement, and time of postoperative recovery.

**Results:**

Compared to the NO-PVB group, the amount of remifentanil used during surgery in patients with TL-PVB group was significantly reduced (1.78 ± 0.37 mg vs. 3.09 ± 0.48 mg, *p* < 0.001), the amount of oxycodone used 24 h after surgery was significantly reduced (8.70 ± 1.70 mg vs. 13.79 ± 2.74 mg, *p* < 0.001), and the amount of oxycodone used 48 h after surgery was remarkably reduced (21.83 ± 4.28 mg vs. 27.27 ± 4.76 mg, *p* < 0.001). There were significant differences in the changes of HR and MAP between the two groups (*p* < 0.001). The first analgesic requirement time of TL-PVB group was significantly longer than that of NO-PVB group (468.56 ± 169.60 min vs. 113.48 ± 37.26 min, *p* < 0.001). The postoperative VAS during rest and movement of TL-PVB group were significantly lower than that of NO-PVB group (*p* < 0.01). Compared with NO-PVB group, patients in TL-PVB group needed shorter time to awaken from anesthesia, leave the operating room, anal exhaust, get out of bed, and had shorter length of postoperative hospital stay (*p* < 0.001). The incidence of postoperative adverse reactions were lower in the TL-PVB group than that in the NO-PVB group (*p* < 0.05).

**Conclusions:**

Ultrasound-guided thoracolumbar paravertebral block significantly reduces intraoperative and postoperative opioid consumption, and provides better analgesia in patients undergoing robot-assisted laparoscopic nephrectomy, which is a recommendable combined anesthesia technique.

**Trial registration:**

ChiCTR2200061326, 21/06/2022.

## Introduction

Robot-assisted laparoscopic nephrectomy overcomes the limitations of traditional laparoscopic surgery, and has the advantages of minimally invasive, short time, quick recovery, and few complications [1, 2]. However, the somatic and visceral pain caused by robot-assisted laparoscopic nephrectomy cannot be ignored. Negatively controlled pain is able to develop into chronic pain, which will affect the prognosis of patients [3, 4]. Continuous epidural technology, nerve block technology, opioid and non-opioid analgesics are widely used in pain management after kidney surgery, among which continuous epidural technology is the most commonly used and has the best effect [4–7].

Paravertebral block is an anesthesia technique with unilateral sensory, motor and sympathetic sympathetic nerve block effects that injecting local anesthetics near the nerve roots in the paravertebral space [8, 9]. Paravertebral block has the same blocking effect to epidural technology, and can effectively reduce the incidence of nausea, emesis and urinary retention. Because it can avoid the impact on the opposite sympathetic nerve, it is easier to maintain stable blood pressure, improve pulmonary function, reduce pulmonary complications, and accelerate postoperative recovery [9, 10]. Paravertebral block is widely used in thoracic surgery, especially cardiothoracic surgery and breast surgery, which is limited in abdominal surgery by the need for bilateral paravertebral block. Previous studies have shown that paravertebral block is used for unilateral kidney surgery, such as percutaneous nephrolithotripsy, nephrectomy, pyeloplasty, etc. It can significantly reduce the pain of patients, reduce the consumption of opioid, and inhibit stress response [11–13].

The abdominal wall is mainly innervated by T6 to L1 nerves [14]. The sensory nerves of the kidney and ureter are transmitted by the nerve fibers of the renal plexus, testicular (ovarian) plexus and lower abdominal plexus, the afferent fibers of these nerves originate from T10 to L2 spinal nerves [15]. The termination of psoas major muscle at T12 vertebral body caused the interruption of thoracic paravertebral space and T12 paravertebral space, and whether the thoracic paravertebral nerve block diffused to T12 and lumbar paravertebral space remains controversial [16–18]. Ozkan et al. dissected 3 cadavers and found that the injection of 15 ml methylene blue at the T10 level did not spread to the T12 and L1 paravertebral side, and at the meantime, the T10 and L1 two-segments paravertebral block had the same effect with the T10 to L1 four-segments paravertebral block [18]. The position of trocars of robot-assisted laparoscopic surgery is farther away and the incision is lower, which requires thoracolumbar paravertebral block, to maintain perfect incision and visceral analgesia.

Therefore, according to the surgical incision and renal innervation, the aim of our study is to select T9 paravertebral combined with L1 paravertebral injection of local anesthetic to complete thoracolumbar paravertebral block and evaluate the analgesic effect in patients undergoing robot-assisted laparoscopic nephrectomy.

## Materials and methods

### Ethical considerations

**Fig. 1:**
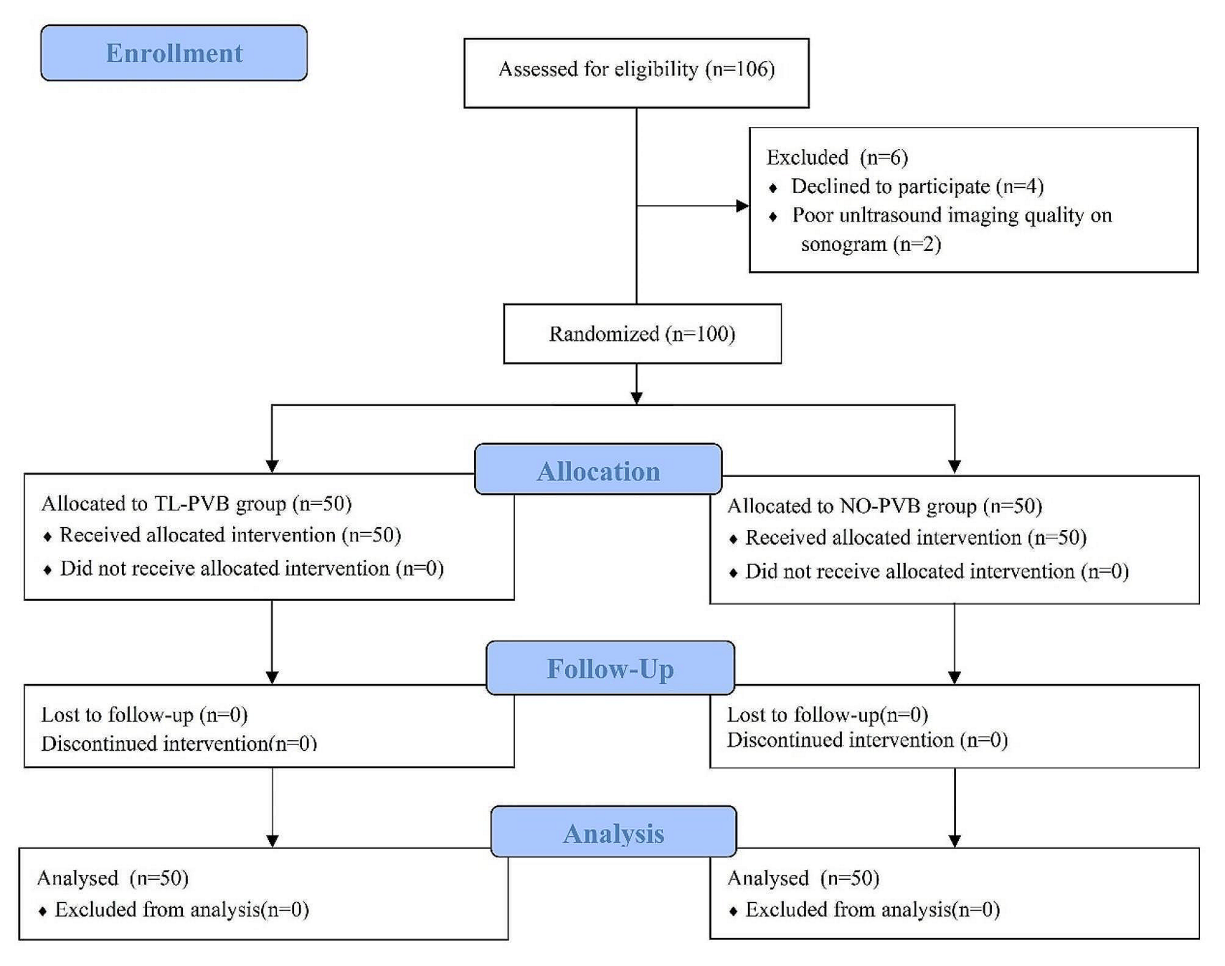
Consolidated standards of reporting trials (CONSORT) flow chart

The randomized controlled trial was approved by the Ethics Committee of General Hospital of Central Theater Command of People’s Liberation Army, China, on May 11, 2022 (reference number:[2022]020 − 01). The trial was registered with the Chinese Clinical Trial Registry (ChiCTR2200061326) on June 21, 2022. It was conducted in accordance with the Consolidated Standards of Reporting Trials (CONSORT) statement and Helsinki declaration (Fig. 1). Written informed consent was obtained from all participants before surgery.

### Patient selection

This prospective, randomized controlled and double-blind trial was conducted in the General Hospital of Central Theater Command of People’s Liberation Army of China. After acquiring written informed consent, 106 patients undergoing elective unilateral robot-assisted laparoscopic nephrectomy between June 21, 2022 and April 21, 2023 were enrolled. Figure 1 presents the study flowchart. Eligibility requirements for inclusion in this study were an aged 18–70 years, a body mass index (BMI) 18–32 kg/m², and an American Society of Anesthesiologists (ASA) classification I-II. We excluded patients who declined to participate in the study, had difficulties in communication, were allergic to local anesthetics, had abnormal coagulation function, were taking anticoagulant drugs, had a history of mental illness or chronic pain, had infection or tumor at the puncture site, had alcohol or long-term drug addiction, and had poor ultrasound imaging quality on sonogram. The researchers publicized the use methods and requirements of PCIA pump, explained the VAS in detail before surgery, to ensure the accuracy of the use of PCIA pump and the evaluation of VAS.

### Study intervention

All patients were assigned to the thoracolumbar paravertebral block combined with general anesthesia (TL-PVB) group and the simple general anesthesia (NO-PVB) group 1:1 according to the computer random number table method and the sealed envelope method. Before the start of the study, an independent person performed allocation randomly by computer-generated number method, and put the numbers into sealed envelopes. Each patient selected an envelope, and was grouped according to the envelope results. The researchers who randomized the grouping did not participate in the follow-up further evaluation. The surgical approach was via the ventral approach and all operations were performed by the same group of surgeons. The chief surgeon has extensive experience in robotic surgery. Trocar insertion was performed by the same first assistant.

Both groups of patients entered the pre-anesthesia room for anesthesia induction. The TL-PVB group was given a single injection of paravertebral block at T9 and L1 after anesthesia induction, and the NO-PVB group was given simple general anesthesia. Both general anesthesia induction and paravertebral block were completed by the same experienced senior anesthesiologist. This anesthesiologist had been engaged in ultrasound-guided nerve block technology for a long time and mastered the paravertebral block technology. After the paravertebral block were completed, wait 10 min to enter the operating room for surgery so that the thoracolumbar paravertebral block could fully took effect during the surgery. To avoid the subjective impact on the intraoperative management, the intraoperative anesthesia management was carried out by another group of senior anesthesiologists who didn’t know the grouping situation. Because the paravertebral block was performed after anesthesia induction and the data collector did not participate in the anesthesia management, neither the data collector nor the patient knew the grouping status. Both groups of patients were treated with oxycodone PCIA pump after surgery, and the prescription and parameters were formulated by experienced anesthesiologists.

### Anesthesia management

After entering the pre-anesthesia room, the patients in both groups were monitored routinely and continuously using the five-lead electrocardiogram, non-invasive blood pressure and pulse oxygen saturation. All patients established a venous access in the upper limb of the non-operative side to infuse sodium acetate Ringer injection, and performed ipsilateral radial artery puncture and catheterization under local anesthesia to monitor arterial blood pressure. Both groups of patients were treated with the same general anesthesia induction and maintenance drugs, received intravenous injection of midazolam (Nhwa Pharmaceutical Co., Ltd, Jiangsu, China) 0.05 mg/kg, etomidate (Nhwa Pharmaceutical Co., Ltd, Jiangsu, China) 0.3 mg/kg, sufentanil (Humanwell Pharmaceutical Co., Ltd, Hubei, China) 0.5 µg/kg, cisatracurium besylate (Hengrui Pharmaceuticals Co., Ltd, Jiangsu, China) 0.3 mg/kg chronologically for anesthesia induction and tracheal intubation. During the operation, propofol (Corden Pharma S.P.A., Caponago, Italy) 4–8 mg/kg/h, remifentanil (Humanwell Pharmaceutical Co., Ltd, Hubei, China) 0.1–0.5 µg/kg/min were continuously pumped, and cisatracurium besylate 0.1 mg/kg was injected intermittently to maintain anesthesia. The bispectral index (BIS) was maintained at 40–60 to keep anesthesia depth, and the blood pressure and heart rate were kept within ± 20% of the base value. When necessary, ephedrine (NORTHEAST PHARM, Shenyang, China) and atropine (Runhong Pharmaceutical Co., Ltd, Henan, China) were used to treat hypotension and bradycardia, nicardipine (Nipro Pharma Corporation Ise Plant, Matsusaka-shi, Japan) was used to treat hypertension. About 30 min before the end of the operation, sufentanil 0.15 µg/kg and flurbiprofen axetil (Tide Pharmaceutical Co., Ltd, Beijing, China) 50 mg were given for preventive analgesia.

### Ultrasound-guided thoracolumbar paravertebral block procedure

**Fig. 2:**
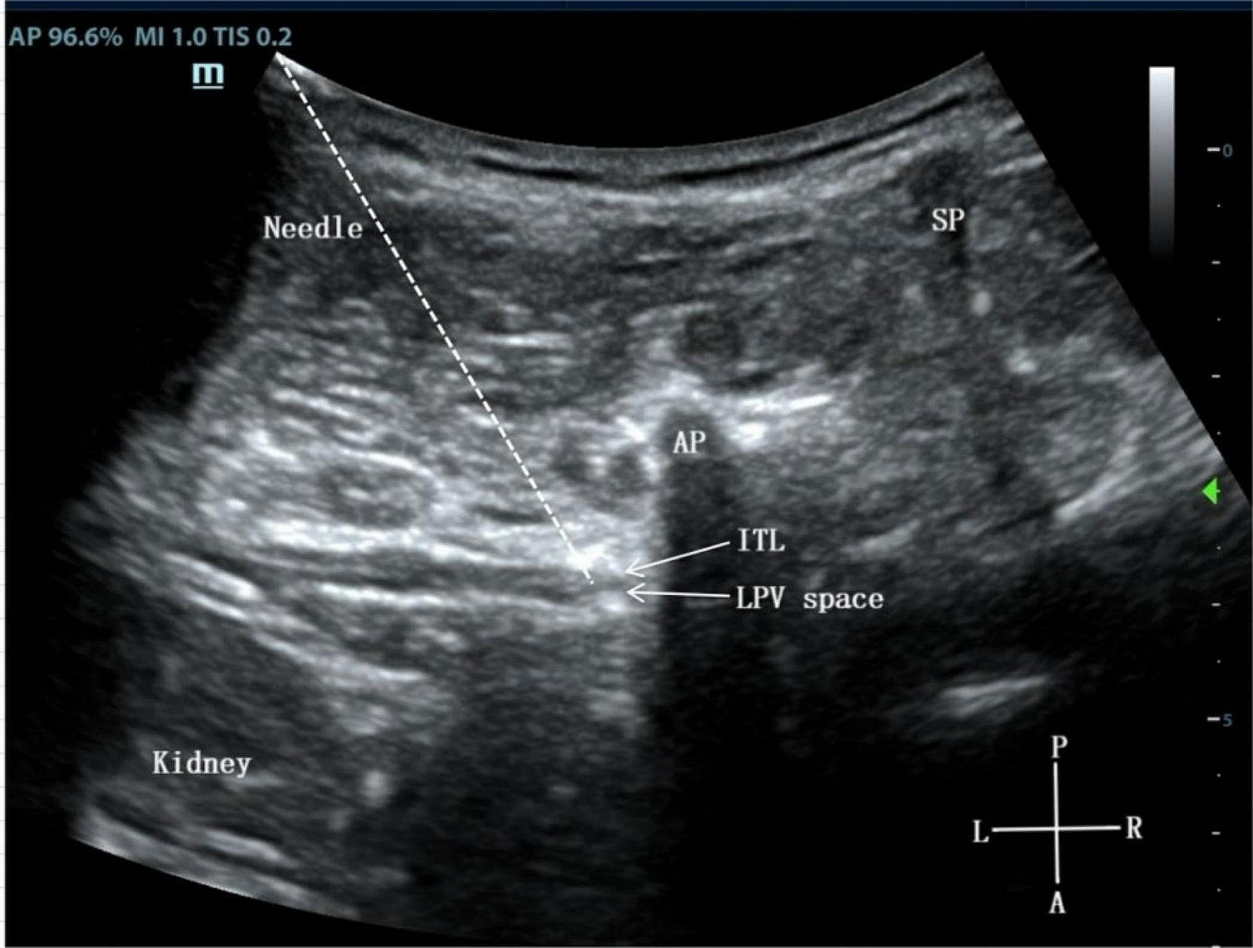
L1 paravertebral block under ultrasound guidance. SP, spinous process; AP, articular process; ITL, intertransverse ligament; LPV space, lumbar paravertebral space; P, posterior; A, anterior; L, left; R, right

After the induction of general anesthesia, the patients in TL-PVB group were placed in a lateral lying position while the operation side was upward, and the sterile towel was draped after conventional disinfection finished, strictly following the aseptic principle. The low-frequency convex array probe of portable ultrasound (M9, Mindray Medical, China) was selected, and it was wrapped with sterile protective cover. Place the ultrasound probe parallel to the paraspinal sagittal position on the sacrum. The sacrum present as a continuous high-echo bone window. As the probe moves toward to the head side, the first transverse process that appears is L5 transverse process. Continue to move the probe toward the head side to locate the L1 transverse process, rotate the probe to the axial position, toward the tail side and avoid the transverse process to locate the L1 paravertebral space, fine-tune the probe and clearly display the spinous process, articular process, intertransverse ligament and lumbar paravertebral space (Fig. 2). Using the in-plane technique, the puncture needle (AN-N 0.7 × 90 mm, Weimao Medical Technology Co., Ltd, Jiangsu, China) was parallel to the probe and inserted from the ventral side, after the puncture needle penetrated the intertransverse ligament, 0.5% ropivacaine (Naropin, AstraZeneca AB Company, Sodertalje, Sweden) 0.1 ml/kg was injected to lumbar paravertebral space [19].

**Fig. 3:**
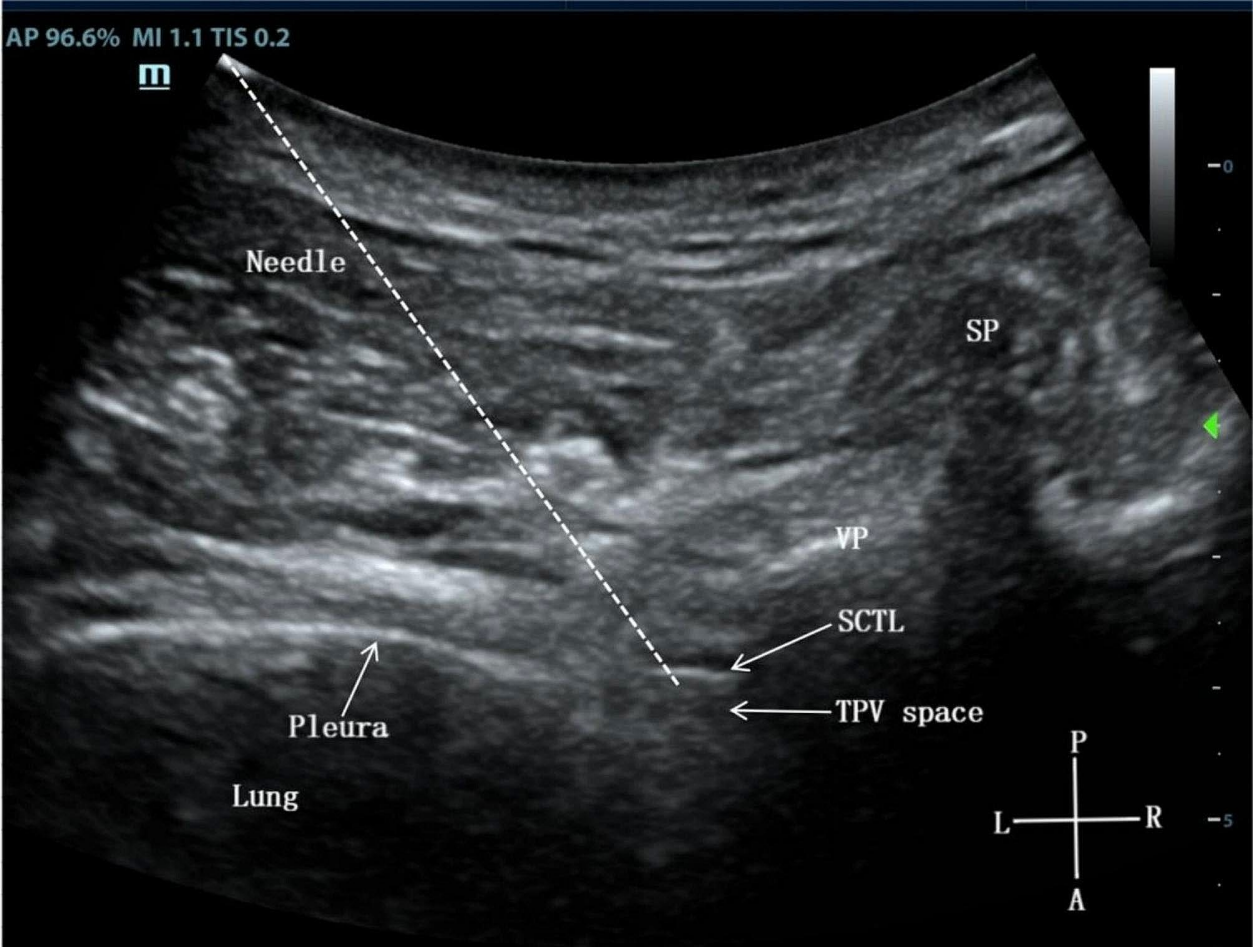
T9 paravertebral block under ultrasound guidance. SP, spinous process; VP, vertebral plate; SCTL, superior costotransverse ligament; TPV space, thoracic paravertebral space; P, posterior; A, anterior; L, left; R, right

Continue to move the probe from the L1 transverse process to the head side to locate the T9 paravertebral space, and adjust the probe parallel to the rib axis to scan the spinous process, vertebral plate, pleura, superior costotransverse ligament and thoracic paravertebral space (Fig. 3). Using the in-plane technique, the needle was inserted from the thoracic side, and 0.5% ropivacaine 0.3 ml/kg was injected to thoracic paravertebral space after the puncture needle penetrated the superior costotransverse ligament [19, 20].

### Postoperative pain management

Postoperatively, all patients were treated with oxycodone (HAMOL LIMITED, NOTTINGHAMSHIRE, U.K.) 0.2 mg/ml for PCIA pump. The bolus was calculated according to oxycodone 0.01 mg/kg, with no background infusion and a 10-min lockout interval. The pain was assessed by the VAS (0, no pain; 1–3, mild pain; 4–6, moderate pain; ≥ 7, severe pain), and the patients were explained about the same. The patients were instructed to use PCIA when the VAS was > 3 at rest, and if the VAS of two consecutive boluses still > 3 at rest, the competent doctor could provide flurbiprofen axetil 50 mg for relief analgesia [11].

### Outcomes assessments

The primary outcomes included the dosage of remifentanil during surgery and the dosage of oxycodone in 24 and 48 h after surgery. The secondary outcomes included the HR and MAP of patients before anesthesia induction (T_0_), when surgery began (T_1_), artificial pneumoperitoneum started (T_2_), surgery finished (T_3_) and leaving the operating room (T_4_), as well as the time of the patient’s first analgesia administration (when VAS > 3), the VAS during rest and movement at 2, 12, 24, 48 h after surgery, the time to awaken from anesthesia, the time to leave the operating room, anal exhaust time, the first time to get out of bed, and the length of postoperative hospital stay. Besides, the complications of paravertebral nerve block, such as pneumothorax, local anesthetic poisoning, hematoma, infection, etc., and the occurrence of nausea, emesis and skin itching caused by opioid drugs were recorded too.

### Sample size calculation and statistical analysis

This study was a randomized controlled trial. The experimental group was a thoracolumbar paravertebral block combined with general anesthesia group (TL-PVB group), and the control group was a simple general anesthesia group (NO-PVB group). Our study was powered using a pilot study to detect the dosage of oxycodone at 48 h after surgery between the TL-PVB group and the NO-PVB group. Pilot study included 20 patients (10 per group) showed a mean oxycodone consumptions of 26.43 mg with standard deviation of 7.42 during 48 h after surgery in the NO-PVB group and 21.52 mg with standard deviation of 6.27 in the TL-PVB group [2]. Using PASS 15 software (NCSS, Kaysville, Utah, USA) to calculate, a minimum number of 86 patients (43 patients per group) was required to reach a study power of 90% and an alpha error of 0.05. Considering the loss and refusal of follow-up, the calculation is based on 10%. Finally, at least 48 subjects in the experimental and control groups were required, and at least 96 subjects should be included.

Data were collected and entered into the computer as numerical or categorical data. SPSS 25.0 Software (IBM Corp, Armonk, NY, USA) and GraphPad Prism 8.0 Software (GraphPad Prism Software Inc., San Diego, California, USA) were used for statistical analysis and graphs generation [11]. Complete descriptive statistics were recorded for each variable, including mean, standard deviation (SD), median (M), and interquartile range (IQR) [15]. The Kolmogorov–Smirnov test was used to determine whether the variables were normally distributed [6]. The independent t-test or Mann–Whitney U test was used for the intergroup comparisons accordingly. The chi-square test or Fisher exact test was used to compare qualitative variables. The repeated measurement data was used repeated measurement analysis of variance. The Kaplan–Meier analysis was performed to assess the time for first analgesia administration. For all comparisons, a *p* value < 0.05 was considered statistically significant, and the differences were then identified.

## Results

Ultimately, one hundred and six patients were enrolled in the study (Fig. 1). Four patients refused to participate in the study. Two patients with poor quality ultrasound imaging were excluded from this study. The remaining 100 patients were randomly allocated into two groups (*n* = 50/group), and none of them dropped out of the study. In the end, the case data of 50 patients in each group were analyzed. There was no significant difference in gender, age, height, weight, BMI, ASA classification, the operative site and duration of surgery between the two groups (*p* > 0.05; Table 1). The two groups were comparable in terms of demographic and clinical characteristics.

**Table 1:** Demographic and clinical characteristics

Variable	TL-PVB(*n* = 50)	NO-PVB(*n* = 50)	*p*-Value
Gender (male/female)	25/25	26/24	0.84
Age (years)	54.78 ± 8.97	55.48 ± 8.77	0.69
Height (cm)	166.54 ± 6.56	165.70 ± 7.30	0.54
Weight (kg)	65.16 ± 8.30	65.24 ± 8.20	0.96
BMI (kg/m^2^)	23.50 ± 2.73	23.70 ± 1.98	0.67
ASA classification (I/II)	16/34	17/33	0.83
Operative site (left/right)	34/16	33/17	0.83
Duration of surgery (min)	164.32 ± 21.82	163.92 ± 23.63	0.93

Numerical variables are expressed as mean ± SD. Categorical variables are expressed as number of patients. BMI, body mass index; ASA, American Society of Anesthesiologists

**Fig. 4:**
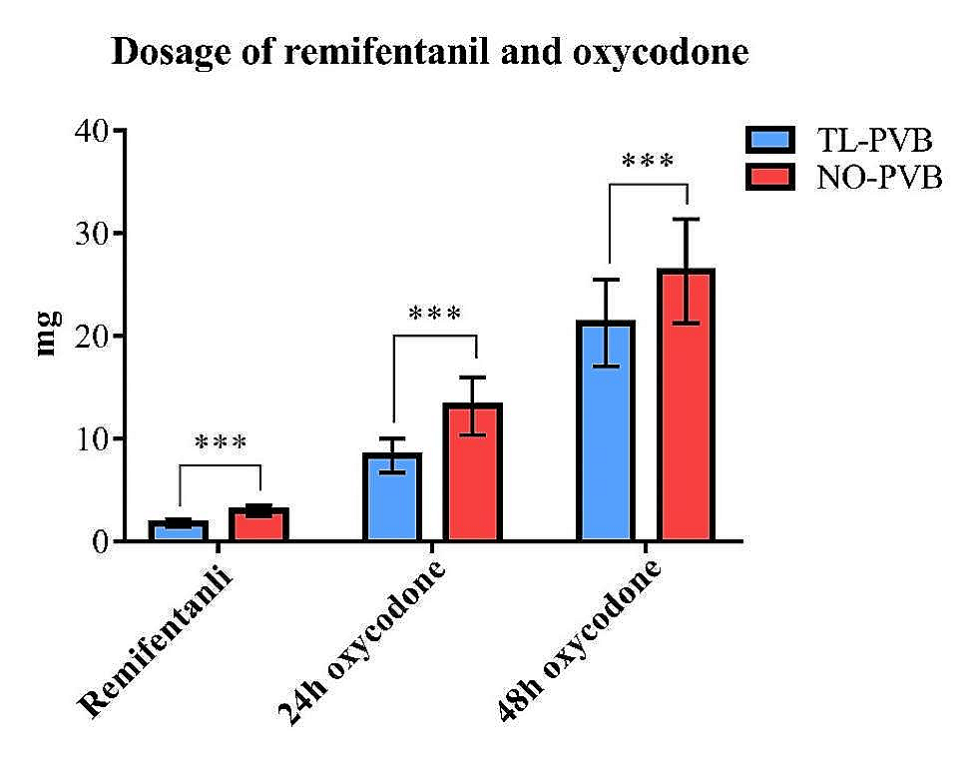
Comparison of intraoperative remifentanil and postoperative oxycodone dosage between the two groups. ***p<0.001

Patients in the TL-PVB group exhibited a significant 42.39% reduction in intraoperative remifentanil use in comparison with patients in the NO-PVB group (1.78 ± 0.37 mg vs. 3.09 ± 0.48 mg, *p* < 0.001; Fig. 4). Compared to NO-PVB group, patients in TL-PVB group required 36.91% less oxycodone use within 24 h after surgery (8.70 ± 1.70 mg vs. 13.79 ± 2.74 mg, *p* < 0.001; Figs. 4) and 19.94% less oxycodone use within 48 h after surgery (21.83 ± 4.28 mg vs. 27.27 ± 4.76 mg, *p* < 0.001; Fig. 4) .

**Fig. 5:**
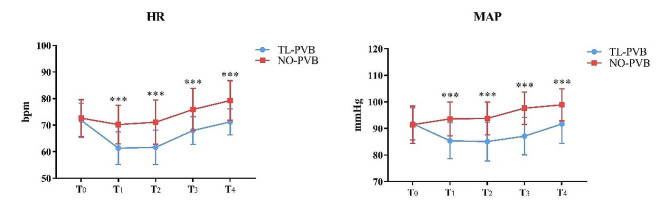
Comparison of HR and MAP between the two groups at different points of time. ***p<0.001. T_0_, before anesthesia induction; T_1_, surgery began; T_2_, artificial pneumoperitoneum started; T_3_, surgery finished; T_4_, leaving the operating room

The results of repeated measures analysis of variance (RM-ANOVA) between the two groups showed that there were significant differences in the intra-group, inter-group and interaction effects of HR and MAP (*p* < 0.001; Fig. 5). The HR and MAP of patients in TL-PVB group were significantly lower than that in the NO-PVB group from T_1_ to T_4_ (*p* < 0.001; Fig. 5).

**Fig. 6:**
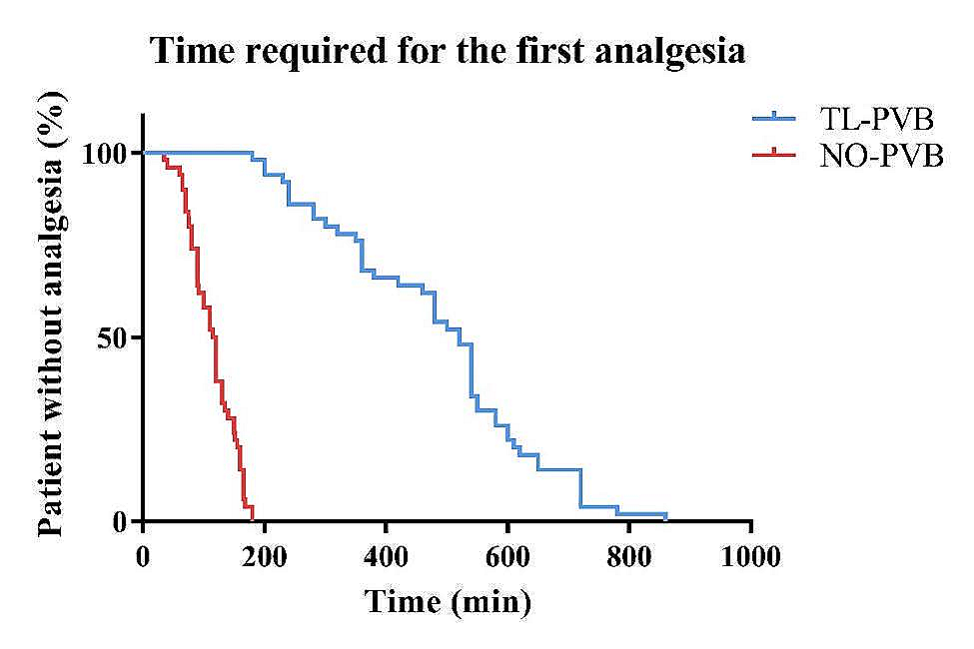
Kaplan-Meier estimate of time to first analgesia. The participant’s ‘survival’ ended with the first PCIA request

**Fig. 7:**
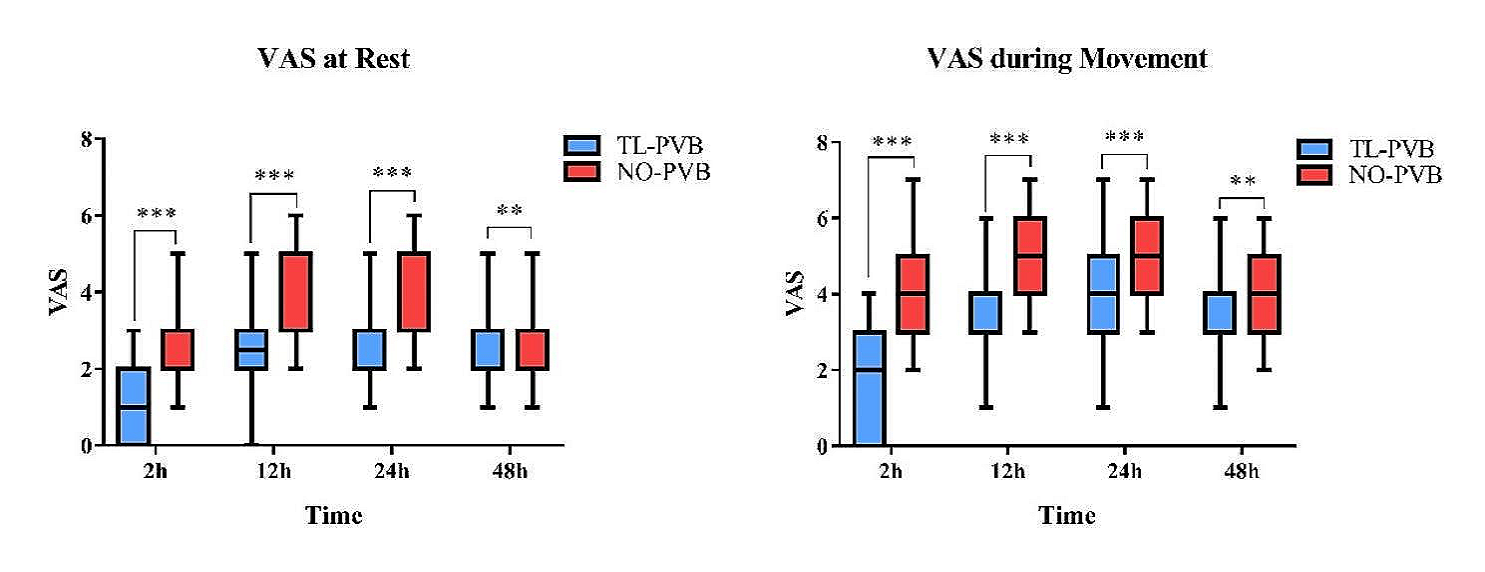
Comparison of VAS at rest and during movement between the two groups at different points time. ***p<0.001; **p<0.01

The time for first analgesia administration in the TL-PVB group was markedly prolonged in comparison with NO-PVB group (468.56 ± 169.60 min vs. 113.48 ± 37.26 min, *p* < 0.001;Fig. 6). The VAS of patients at rest and during movement were much lower in TL-PVB group than those in NO-PVB group (*p* < 0.01; Fig. 7).

The time to awaken from anesthesia, time to leave the operating room, anal exhaust time, first time to get out of bed, and the length of postoperative hospital stay in TL-PVB group were significantly shortened than NO-PVB group (*p* < 0.001; Table 2). There were no complications such as hematoma, infection, pneumothorax or nerve injury in the patients of the TL-PVB group. Compared with NO-PVB group, the incidence of adverse reactions of TL-PVB group decreased (4 cases vs. 12 cases, *p* < 0.05; Table 2). There were 3 patients with dizziness, 3 patients with nausea, and 2 patients with emesis in TL-PVB group. There were 4 patients experienced dizziness, 9 patients experienced nausea, and 6 patients experienced emesis in NO-PVB group.

**Table 2:** Postoperative recovery time and adverse reactions

Variable	TL-PVB(*n* = 50)	NO-PVB(*n* = 50)	*p*-Value
Anesthesia awaken time(min)	9.28 ± 4.58	17.68 ± 8.51	*p* < 0.001
Time to leave the operating room (min)	39.18 ± 4.67	50.22 ± 8.82	*p* < 0.001
Anal exhaust time(day)	1.0 (1.0–2.0)	2.0 (2.0–2.0)	*p* < 0.001
First time to get out of bed(day)	1.0 (1.0–2.0)	2.0 (2.0–3.0)	*p* < 0.001
Length of postoperative hospital stay(day)	7.0 (7.0–8.0)	8.5 (7.0–10.0)	*p* < 0.001
Incidence rate of adverse reactions	4 (8%)	12 (24%)	0.029
Dizziness	3 (6%)	4 (8%)	0.695
Nausea	3 (6%)	9 (18%)	0.065
Emesis	2 (4%)	6 (12%)	0.140

Numerical variables are expressed as mean ± SD. Categorical variables are expressed as number of patients (percentage of patients)

## Discussion

Renal cancer is the disease with the highest mortality rate caused by the cancer of the urinary and reproductive system. Just in 2020, 431,288 new cases of renal cancer were found in the world, with its incidence rate ranking 14th in malignant tumors, 9th and 14th in male and female malignant tumors respectively, and showing an increasing trend year by year [21]. Surgical resection is the main method for treating renal tumors. With advantages such as three-dimensional high-definition visual field, tremor filtering function, and flexible instrument operability, robot-assisted surgery systems have overcome the shortcomings of traditional laparoscopic techniques, and are increasingly being used in urological surgery for nephrectomy [22, 23]. However, robot-assisted laparoscopic nephrectomy generally combines somatic pain caused by surgical incisions, inflammatory pain caused by noxious stimuli, and visceral pain caused by chemical and mechanical stimulation [5, 24]. The mechanism of pain is complex, and perioperative pain control is extremely important.

At present, the analgesic effect of thoracolumbar paravertebral block in patients undergoing robot-assisted laparoscopic nephrectomy is unclear. The main purpose of our study was to observe whether thoracolumbar paravertebral block can reduce the amount of intraoperative remifentanil used and postoperative oxycodone used in patients undergoing robot-assisted laparoscopic nephrectomy, and to improve the postoperative analgesic effect. This study is different from previous studies in that it is the first time to observe the effect of thoracolumbar paravertebral block combined with general anesthesia on intraoperative and postoperative analgesia in patients undergoing robot-assisted laparoscopic nephrectomy. Saito et al. found that 10 ml of local anesthetics can block five segments of thoracic vertebrae by single-segment paravertebral injection [25]. Thoracolumbar paravertebral block was selected based on the innervation of the kidney and surgical incision location, and related operations were performed under ultrasound guidance. Based on past experiences and researches, local anesthetic injection doses were set to ensure effectiveness while avoiding complications [15, 18, 25]. The termination of the psoas major muscle at the T12 vertebral body resulted in a lack of communication between the thoracic paravertebral space and the lumbar paravertebral space. Simply injecting local anesthetics into the thoracic paravertebral space may not block the T12 and lumbar paravertebral nerves. Therefore, in order to achieve a complete analgesic effect, we selected T9 and L1 to establish paravertebral block, based on the anatomical structure, studies and application results in previous.

In our study, the amount of remifentanil used in the TL-PVB group was 42.39% lower than that in the NO-PVB group during surgery (Fig. 4), the amount of oxycodone used 24 h after surgery was 36.91% lower (Fig. 4), and the amount of oxycodone used 48 h after surgery was 19.94% lower (Fig. 4), which indicated that thoracolumbar paravertebral block can significantly reduce the amount of opioids used during and after robot-assisted laparoscopic nephrectomy. The research results of Copik et al. showed that patients receiving thoracic paravertebral block combined with general anesthesia during open nephrectomy had a 39% reduction in the need for intravenous oxycodone within 48 h after surgery compared to patients receiving simple general anesthesia, with reduced postoperative pain and adverse opioid events, and it was coincident with our study [26]. Compared to this study, the difference in the reduction rate of oxycodone may be related to different paravertebral block schemes, surgical methods, and population differences.

Thoracic paravertebral block has a unilateral epidural effect and does not affect the contralateral sympathetic nerve, which provides more stable hemodynamics [8–10]. In addition, Tang et al. compared the application of thoracic paravertebral block combined with general anesthesia and simple general anesthesia in patients undergoing laparoscopic radical nephrectomy [11]. The results showed that patients receiving thoracic paravertebral block combined with general anesthesia had lower heart rate and blood pressure, lower VAS during rest and movement 48 h after surgery, and fewer adverse reactions such as nausea and emesis than patients undergoing simple general anesthesia. Similarly, in our study, the HR and MAP of patients in TL-PVB group were lower than those in NO-PVB group at the beginning of surgery, at the time when artificial pneumoperitoneum started, at the end of surgery, and at the time of leaving the operating room (Fig. 5), indicating that thoracolumbar paravertebral block combined with general anesthesia can reduce the hemodynamic effects of surgical stimulation.

In a similar study, Baik et al. demonstrated that thoracic paravertebral block can reduce postoperative fentanyl use and VAS at various time points 24 h after surgery in patients undergoing open nephrectomy, and thereby improve postoperative analgesia [27]. By comparing the postoperative analgesic effects, it was found that patients in TL-PVB group had an average prolongation of 355.08 min in their first postoperative analgesic need compared to patients in NO-PVB group in our study (Fig. 6), and their VAS during rest and movement at various time points after surgery was significantly reduced (Fig. 7), which further demonstrated that thoracolumbar paravertebral block can reduce the amount of opioids and improve the postoperative analgesic effect. The above results of our study indicated that thoracolumbar paravertebral block can reduce the amount of opioids used during and after surgery, decrease the hemodynamic effects of surgical stimulation, and provide better analgesic effects.

Previous studies have shown that over 80% of patients had moderate to severe postoperative pain, and up to 70% of patients still had significant pain even after discharge [28]. Poorly controlled pain could lead to slowly postoperative recovery, wound infection, increased risk of cardiovascular complications, and delayed discharge [29, 30]. Perioperative weak opioid therapy can not only reduce postoperative adverse reactions and complications, but also have positive significance for tumor prognosis [28, 31]. In our study, it was confirmed that thoracolumbar paravertebral block combined with general anesthesia can reduce the amount of opioids used during and after surgery, and improve the postoperative analgesic effect. At the same time, it was observed that the postoperative anesthesia awaken time, the time to leave the operating room, the time to anal exhaust, the first time to get out of bed, and the length of postoperative hospital stay were shorter than those of simple general anesthesia, indicating that thoracolumbar paravertebral block can accelerate the postoperative recovery of patients.

In this study, there were no adverse reactions and complications related to the procedure in TL-PVB group, confirming the safety of ultrasound-guided thoracolumbar paravertebral block. It also reduced postoperative opioid related adverse reactions, which may be related to less use of opioids.

Some limitations can be found in our study. Firstly, the NO-PVB group has not been subjected to saline control and has not been compared with other blocking methods, hence it can only demonstrate that the effect is superior to that of patients under simple general anesthesia. Secondly, this study is a single center study, lacking of multicenter large sample studies. Finally, the long-term recovery effect of the patients was not observed. These limitations can be consummated by further research in the future.

## Conclusions

The results of our study indicated that thoracolumbar paravertebral block combined with general anesthesia is more suitable for robot-assisted laparoscopic nephrectomy than simple general anesthesia, and can provide better intraoperative and postoperative analgesic effects. Thoracolumbar paravertebral block is a better combined anesthesia technique, which can reduce intraoperative and postoperative opioid dosage, decrease the hemodynamic effects of surgical stimulation, and reduce postoperative VAS, thus extending the time required for initial analgesia and accelerating the recovery of patients after surgery.

## Data Availability

The datasets used and analysed during the current study not publicly available due to ethical reasons but are available from the corresponding author on reasonable request.
